# Resistance to Bt Maize by Western Corn Rootworm: Effects of Pest Biology, the Pest–Crop Interaction and the Agricultural Landscape on Resistance

**DOI:** 10.3390/insects12020136

**Published:** 2021-02-05

**Authors:** Aaron J. Gassmann

**Affiliations:** Department of Entomology, Iowa State University, Ames, IA 50011, USA; aaronjg@iastate.edu

**Keywords:** dispersal, field-evolved resistance, fitness costs, inheritance, integrated pest management, pyramid strategy, refuge strategy, resistance management

## Abstract

**Simple Summary:**

Since the 1990s, an important innovation in the management of agricultural pest insects has been the commercial cultivation of genetically engineered crops that produce insecticidal toxins, which in turn act to protect plants from feeding injury by insects. To date, these transgenic crops, which include cotton, maize and soybean, have produced insecticidal proteins derived from the bacterium *Bacillus thuringiensis* (Bt). Benefits associated with planting of Bt crops include reduced feeding injury from pest insects, decreased yield losses from pests and less harm to the environment. However, the evolution of Bt resistance by insect pests can diminish these benefits. One serious insect pest currently managed with Bt maize is the western corn rootworm. The larval stage of this insect feeds on maize roots and can substantially reduce yield. In some parts of the US Corn Belt, western corn rootworm rapidly adapted to Bt maize, and currently, some populations show resistance to all commercially available Bt traits. This review summarizes the time course of resistance development in the field, key factors contributing to resistance evolution, and steps that biotechnology companies, farmers and regulatory agencies can take to delay additional cases of pest resistance to current and future transgenic technologies.

**Abstract:**

The western corn rootworm, *Diabrotica virgifera virgifera* LeConte, is among the most serious pests of maize in the United States. Since 2003, transgenic maize that produces insecticidal toxins from the bacterium *Bacillus thuringiensis* (Bt) has been used to manage western corn rootworm by killing rootworm larvae, which feed on maize roots. In 2009, the first cases of field-evolved resistance to Bt maize were documented. These cases occurred in Iowa and involved maize that produced Bt toxin Cry3Bb1. Since then, resistance has expanded to include other geographies and additional Bt toxins, with some rootworm populations displaying resistance to all commercially available Bt traits. Factors that contributed to field-evolved resistance likely included non-recessive inheritance of resistance, minimal fitness costs of resistance and limited adult dispersal. Additionally, because maize is the primary agricultural crop on which rootworm larvae can survive, continuous maize cultivation, in particular continuous cultivation of Bt maize, appears to be another key factor facilitating resistance evolution. More diversified management of rootworm larvae, including rotating fields out of maize production and using soil-applied insecticide with non-Bt maize, in addition to planting refuges of non-Bt maize, should help to delay the evolution of resistance to current and future transgenic traits.

## 1. Introduction

Planting of transgenic maize that produces insecticidal toxins derived from the bacterium *Bacillus thuringiensis* (Bt) has played a prominent role in the management of western corn rootworm, *Diabrotica virgifera virgifera* LeConte, for nearly two decades. The release of the first transgenic events for management of corn rootworm followed several years of successful management of some other key insect pests of maize and cotton [1,2]. However, within six years of the initial release of Bt maize targeting western corn rootworm, the first cases of field-evolved Bt resistance were observed [3]. Since then, field-evolved resistance to all available Bt traits has been documented, and resistance to some Bt traits appears to be widespread within certain regions of the US Corn Belt [4,5,6,7]. The goals of this paper are to review the time course of field-evolved resistance to Bt maize by western corn rootworm, discuss the factors associated with the evolution of resistance, and consider how the management of western corn rootworm could be improved for current and future transgenic technologies. 

The western corn rootworm is one of the most serious pests of maize in the United States [8]. Most yield losses associated with this pest are from larval feeding on maize roots [9,10,11]. In the United States, annual economic losses associated with corn rootworm, including both management costs and yield losses, range between $1 to $2 billion [12]. Western corn rootworm is a univoltine pest and its primary larval host is maize [13]. As such, fields that are planted to maize for several consecutive years provide the ideal habitat for this pest, and can be associated with large populations of western corn rootworm and high levels of larval feeding injury [9]. 

## 2. Bt Maize and Resistance Management

Transgenic Bt maize has been used to manage corn rootworm since 2003. The first Bt maize produced a single Bt trait, Cry3Bb1, and subsequently three addition Bt traits were registered by the US Environmental Protection Agency (EPA): Cry34Ab1/Cry35Ab1 (now called Gpp34Ab1/Tpp35Ab1 [14]) in 2005, mCry3A in 2006 and eCry3.1Ab in 2012 [15,16,17,18]. Similar to Cry3Bb1, both Cry34/35Ab1 and mCry3A were used initially as single Bt traits targeting corn rootworm, while eCry3.1Ab was released as a pyramid with mCry3A [19]. Additionally, pyramids of Cry3Bb1 with Cry34/35Ab1 and mCry3A with Cry34/35Ab1 were registered by the US EPA in 2009 and 2012, respectively [20]. Replacement of single Bt traits targeting corn rootworm by pyramids of two Bt traits was a gradual process, and for multiple growing seasons, both single traits and pyramids occurred together in the agricultural landscape [21,22]. Moreover, resistance to some of these Bt traits, in particular Cry3Bb1 and mCry3A, was already present in the agricultural landscape prior to the release of pyramided events [3,23]. 

In the US, an insect resistance management (IRM) strategy is mandated by the US EPA for the commercial cultivation of any Bt crop, including Bt maize that targets corn rootworm [19]. Currently, EPA-mandated IRM approaches for Bt crops focus on the refuge strategy. Under the refuge strategy, a non-Bt host is provided for an insect pest, with the goal of producing Bt-susceptible individuals that can mate with any Bt-resistant individuals surviving on a Bt crop [24]. The use of refuges to delay resistance can be especially effective when combined with either high-dose Bt events or Bt crops that are pyramided with multiple Bt toxins targeting the same pest [25,26,27,28]. 

A high-dose Bt crop is defined as one that either produces 25 times more Bt toxin than necessary to kill a Bt-susceptible pest or kills 99.99% of susceptible individuals [29]. When a high dose is achieved, Bt resistance is rendered a functionally recessive trait because the dose of toxin produced is sufficient to kill not only homozygous susceptible individuals, but also heterozygous resistant individuals [28]. Thus, in the high-dose/refuge scenario, susceptible individuals from a refuge mate with resistant individuals surviving on a Bt crop, producing heterozygous progeny that are unable to survive on a high-dose Bt crop, which in turn delays resistance. 

By contrast, a pyramid delays resistance through redundant killing, with insects that harbor alleles for resistance to one Bt toxin in a pyramid killed by the second toxin and vice versa [30]. In the pyramid/refuge scenario, susceptible individuals from a refuge mate with resistant individuals from a Bt crop, thereby reducing the proportion of individuals that harbor resistance alleles for both Bt toxins in a pyramid, and consequently delaying the evolution of resistance. However, for a pyramid to work effectively, there must be an absence of cross-resistance between the toxins, and alleles for resistance to either toxin must be at a low frequency within the population [31,32]. If a pest population has evolved resistance to one toxin in a pyramid, the delay in resistance achieved by combining two toxins in a pyramid will be greatly diminished or lost altogether [30,32]. 

A critical factor affecting how quickly a pest will evolve Bt resistance is the inheritance of resistance, and this is especially important when Bt traits are deployed singly or if one of two traits in a pyramid is compromised by resistance [32,33]. When resistance to a Bt trait is inherited in a non-recessive manner, some proportion of the heterozygous resistant individuals will not be killed by that Bt trait, and resistance will evolve faster than when resistance is recessive [33]. Furthermore, the rate of resistance evolution is expected to show a positive relationship with the genetic dominance of resistance, and occur at a faster rate as the fitness of heterozygous resistant individuals on a Bt crop increases [27,33]. Thus, understanding the inheritance of resistance is essential for predicting the success of the refuge strategy to delay resistance [27].

Additionally, whether or not Bt resistance has accompanying fitness costs also will affect the rate of resistance evolution [34]. Fitness costs arise, in the absence of Bt, when individuals with resistance alleles have lower fitness than Bt-susceptible individuals [34]. Fitness costs of Bt resistance impose a counter-acting selective force that removes resistance alleles from refuge populations and delays the rate of pest adaptation to a Bt crop [35,36,37]. However, if fitness costs of resistance are minimal or absent, resistance is expected to evolve more rapidly than when fitness costs are present [21]. As such, quantifying the extent to which fitness costs accompany Bt resistance is another key factor in determining whether or not a population will evolve resistance, and how rapidly resistance will evolve and spread.

## 3. Time Course and Current Status of Field-Evolved Resistance 

In 2009, farmers in Iowa observed high levels of feeding injury by western corn rootworm to maize producing Cry3Bb1 [3]. Subsequent plant-based bioassays found that this feeding injury was associated with Cry3Bb1 resistance by western corn rootworm [3]. Additional cases of Cry3Bb1 resistance were identified in Iowa in 2010 [38]. In 2011, field populations were sampled from several fields that had high levels of feeding injury from western corn rootworm to either Cry3Bb1 maize or mCry3A maize, and results of plant-based bioassays revealed resistance to both mCry3A and Cry3Bb1, and cross-resistance between these Bt toxins [23]. In 2012, field populations were sampled across the northern half of Iowa from fields, where high levels of feeding injury to Cry3Bb1 maize were observed, and plant-based bioassays demonstrated that cross-resistance between Cry3Bb1 and mCry3A also extended to eCry3.1Ab [39]. 

Fields that were sampled in these studies were typified by a node or more of feeding injury to roots of Bt maize, and in some cases, more than two nodes, with each node of roots lost translating to a 15% to 17% reduction in yield [10,11]. This means that Bt resistance by western corn rootworm has practical significance for farmers by substantially reducing yield. It is noteworthy that field populations evaluated from 2009 to 2012 were not resistant to Cry34/35Ab1 [3,23,38,39]. However, resistance to the Cry3 traits meant that the IRM advantage of pyramiding had been greatly reduced when Cry34/35Ab1 was placed in a pyramid with a Cry3 trait.

Resistance to Cry3 maize by western corn rootworm is not limited to Iowa and has been documented in Illinois, Minnesota, Nebraska and North Dakota [40,41,42,43]. All four states have areas of intensive maize production, with fields commonly planted to maize for several consecutive years [44]. Additionally, some of these populations were tested for cross-resistance to other Bt traits, and similar to research from Iowa, cross-resistance was found among Cry3Bb1, mCry3A and eCry3.1Ab [40,41]. Field-evolved resistance in some of these states appears to have occurred at a similar time as the observation of Bt resistance in Iowa, suggesting that Bt resistance evolved independently in several locations throughout the Corn Belt. 

The presence of cross-resistance among Cry3Bb1, mCry3A and eCry3.1Ab may be due to the structural similarities among these three-domain Bt toxins, such that genetic changes conferring resistance to one of the Cry proteins likely confer resistance to the others [23,31,39]. By contrast, Cry34/35Ab1 is a binary toxin and differs structurally from three-domain toxins, and thus also likely has a mechanism of toxicity that is independent of the Cry3 toxins [31,39]. To date, little is known about the mechanistic basis of Bt resistance in western corn rootworm [14]. However, the potential for western corn rootworm to develop Cry34/35Ab1 resistance appears similar to that of Cry3Bb1. For example, laboratory selection experiments have shown that western corn rootworm developed Cry34/35Ab1 resistance after three to seven generations of selection [45,46], which is similar to past selection studies with Cry3Bb1 maize [47,48]. 

As suggested by these laboratory selection experiments, following field-evolved resistance to Cry3 maize, studies provided evidence that resistance to Cry34/35Ab1 had emerged in field populations of western corn rootworm. In 2013, fields in Iowa were sampled where high levels of feeding injury from western corn rootworm were observed for Cry34/35Ab1 maize (>2 nodes of injury on average) and for maize pyramided with Cry34/35Ab1 and a Cry3 toxin (>1 node of injury on average). Plant-based bioassays with progeny of western corn rootworm from these fields showed elevated survival on Cry34/35Ab1 maize compared to Bt-susceptible controls, which indicated that the high levels of feeding injury observed in the field were associated with western corn rootworm resistance to Cry34/35Ab1 [49]. However, for these populations, larval survival was lower on Cry34/35Ab1 maize than on non-Bt maize, indicating that resistance was incomplete [49]. Similarly, a field population of western corn rootworm from Minnesota, which was sampled in 2013, was found to have incomplete resistance to Cry34/35Ab1 [50]. 

More recently, Gassmann et al. [51] examined field populations sampled in Iowa during 2017, which were collected from two fields with a high level of western corn rootworm feeding injury to maize pyramided with Cry3 and Cry34/35Ab1. Both populations were found to have resistance to Cry34/35Ab1 in addition to resistance to all three Cry3 toxins. For one population, no difference in larval survival or development was detected between non-Bt maize and maize with Cry34/35Ab1, either alone or in a pyramid with Cry3Bb1, suggesting complete resistance to Cry34/35Ab1. In addition to these field populations associated with injury to Bt maize, Gassmann et al. [51] also included three field populations that were not associated with injury to Bt maize, and one of these populations also displayed resistance to Cry34/35Ab1. Taken together, these data suggest that resistance to Cry34/35Ab1 has persisted in the agricultural landscape, and appears to be increasing in magnitude. A key factor affecting the future utility of Bt maize for management of western corn rootworm will be how quickly additional cases of resistance to Cry34/35Ab1 evolve. 

## 4. Factors Affecting Resistance Evolution

Several factors likely contributed to the rapid development of field-evolved resistance to Bt maize by western corn rootworm. Limited movement of adult rootworm prior to mating and after mating likely reduced the effectiveness of refuges to delay resistance and enabled resistance to build within populations. The lack of a high dose for Bt toxins that target corn rootworm, and the resulting non-recessive inheritance of resistance, coupled with standing genetic variation for resistance within populations, facilitated rapid resistance evolution when rootworm populations were exposed to Bt maize. Additionally, the presence of minimal fitness costs of resistance also favored rapid resistance evolution. 

It appears that adult western corn rootworm engages in limited dispersal within the agricultural landscape, and this likely contributed to field-evolved resistance in multiple ways. Available data suggest that the majority of adults only move about 40 m per day [52,53]. Furthermore, newly emerged, teneral females will often mate near the plant where they emerge [53,54]. As a result, when refuges are spatially structured, with blocks of Bt maize and non-Bt maize, there will be limited mating between Bt-selected individuals and refuge individuals, which in turn will reduce the ability of refuges to delay resistance. When Bt maize was initially released in 2003, only structured refuges were used, with integrated, or blended refuges first planted in 2011. Additionally, available data suggest limited compliance by farmers in the planting of structured refuges, an effect that is expected to further increase the rate of resistance evolution [33,55]. 

Another important consequence of limited adult dispersal is the role of local, within-field selection in driving resistance evolution. Studies of other species of pest insects indicate that limited dispersal can increase the rate of resistance evolution [56]. The western corn rootworm is a univoltine pest, with females mostly laying eggs in maize fields, and eggs then diapausing through the winter and hatching the following spring [9]. Because the primary host for western corn rootworm larvae is maize, continuous maize cultivation is necessary for populations to persist within a field. Limited adult dispersal means that most adult females emerging from a maize field will also oviposit in the same field. Consequently, continuous planting of maize containing the same rootworm trait leads to continuous selection for resistance. The first cases of Cry3Bb1 resistance were associated with continuous cultivation of Cry3Bb1 maize, and there was a positive correlation between the years that a field was planted to Cry3Bb1 maize and the level of Cry3Bb1 resistance [3]. As such, continuous maize cultivation and continuous use of the same Bt trait within a field appears to be an important factor affecting the rate of resistance evolution. This finding is concordant with laboratory selection experiments, which found that resistance to Bt maize can develop rapidly (e.g., within three generations) under continuous laboratory selection in the absence of refuges [45,46,47,48,57,58]. Importantly, the rapid evolution of resistance to Bt maize by western corn rootworm under laboratory selection was found for all currently available Bt traits (i.e., Cry3Bb1, eCry3.1Ab, mCry3A and Cry34/35Ab1). 

One factor that likely contributed to the rapid evolution of resistance in these laboratory selection experiments, and in the field, is the initial frequency for resistance traits within populations. Onstad and Meinke [59] conducted a retrospective analysis of laboratory selection experiments described in Meihls et al. [47] and Lefko et al. [46] and concluded that the initial resistance allele frequency was in the range of 0.05 to 0.20. This is a higher frequency than was found for Bt-resistance alleles in several lepidopteran pests [33], or typically used in simulation models of pest resistance [60,61]. As a result of this higher resistance allele frequency, the rate of evolution is expected to be faster than would occur at lower resistance allele frequencies [28].

A second factor facilitating the development of resistance, in both the laboratory and field, is the inheritance of resistance. Because none of the Bt traits available for management of western corn rootworm produce a high dose of toxin, theory predicts that the inheritance of resistance traits will be non-recessive [21,28,62]. Studies on the inheritance of Bt resistance by western corn rootworm have used both laboratory-selected strains and strains with field-evolved Bt resistance (Table 1). Studies of strains with laboratory-selected Cry3Bb1 resistance found evidence of non-recessive inheritance [47,63]. Similarly, resistance to mCry3A and eCry3.1Ab in laboratory-selected strains was found to be non-recessive, and in some cases dominant [58,64].

Research on strains with field-evolved resistance to Cry3Bb1 maize also found evidence of non-recessive inheritance resistance [65,66,67] (Table 1). In three of four strains, where field-evolved resistance had been introgressed into a non-diapausing background, resistance to Cry3Bb1 maize was found to be non-recessive [65,66]. Additionally, in a study using diapausing western corn rootworm strains with field-evolved resistance to Cry3Bb1 maize, the general pattern was for resistance to be non-recessive, with three of four strains displaying non-recessive inheritance in plant-based bioassays [67].

In the presence of non-Bt refuges, fitness costs of Bt resistance can act to delay the evolution of resistance [34]. Several studies have tested for fitness costs of Bt resistance in strains of western corn rootworm with laboratory-selected resistance and in strains with field-evolved resistance (Table 2). Strains with laboratory-selected resistance to Cry3Bb1 maize have displayed fitness costs in some cases [68,69] but not in others [63,70,71]. Additionally, fitness costs appeared to be absent in strains with laboratory-selected resistance to eCry3.1Ab and mCry3A [58,72].

Patterns of fitness costs associated with field-evolved Cry3Bb1 resistance were similar to results for strains with laboratory-selected resistance, with costs present in some cases but not in others. However, costs appear to be more common in strains with field-evolved resistance compared to laboratory-selected resistance (Table 2). Ingber and Gassmann [65] identified costs affecting larval development, survival to adulthood and fecundity in one strain (Cresco) but costs were absent in another strain (Hopkinton). In a study of two additional strains, Paolino and Gassmann [66] found a fitness cost in one strain (Elma) but not in a second strain (Monona). Shrestha and Gassmann [67] studied several field populations and detected a negative relationship between adult size and the level of Cry3Bb1 resistance, indicating a fitness cost of resistance affecting adult size. St. Clair et al. [73] assessed fitness costs of Cry3Bb1 resistance in Hopkinton and Cresco through a selection experiment, which tested for a loss of resistance over time in the absence of exposure to Cry3Bb1 maize, and this study found evidence of fitness costs in both strains. The contrasting results between Ingber and Gassmann [65], which measured individual life-history characteristics, and St. Clair et al. [73], which used a selection experiment, likely arose because selection experiments provide a more comprehensive metric for assessing fitness costs and are therefore more sensitive [34]. 

However, it is important to note that St. Clair et al. [73] also found that Cry3Bb1 resistance persisted for at least six generations in the absence of exposure to Cry3Bb1 maize, which translates to 6 years in the field, because western corn rootworm has one generation per year. Consequently, to the extent that fitness costs do accompany Cry3Bb1 resistance, it is likely that Bt resistance currently present in the agricultural landscape may remain for several years, even if farmers were to discontinue planting of Bt maize [4,5,6,7]. 

Taken together, the available data suggest that minimal fitness costs may often be associated with Bt resistance in western corn rootworm, and consequently, fitness costs may do little to delay the evolution of Bt resistance. In general, fitness costs tend to increase with the magnitude of resistance, with strains that have higher resistance ratios incurring more fitness costs than strains with lower resistance ratios [34]. Data from strains with field-evolved resistance to Cry3Bb1 maize indicate that resistance ratios tend to range from 2.5 to 19, which is substantially lower than resistance ratios found for pests targeted by high-dose Bt crops [65,66,67]. For example, in cases where a high dose was achieved, such as with Bt cotton that targets pink bollworm *Pectinophora gossypiella* and Bt maize that targets European corn borer *Ostrinia nubilalis*, resistance ratios were greater than 500 [74,75]. As such, for western corn rootworm, fitness costs of resistance to Bt maize may often be less than costs associated with Bt crops that are high dose and require pests to have a much higher resistance ratio to survive [23]. Consequently, fitness costs, and the corresponding delay in resistance evolution, are expected to be less for western corn rootworm than for other pests targeted by Bt crops where a high dose is achieved. Furthermore, fitness costs of Bt resistance in western corn rootworm appear insufficient to delay resistance development in the field, at least with the current refuge requirements. 

Data are currently lacking on the inheritance and fitness costs of resistance to Cry34/35Ab1, in either laboratory-selected strains or in strains with field-evolved resistance. With the emergence of field-evolved resistance to Cry34/35Ab1 [49,50,51], such data would enable valuable insights into how quickly resistance will develop in the broader agricultural landscape. In the case of resistance to Cry3Bb1 maize, which in turn confers cross-resistance to mCry3A and eCry3.1Ab, it appears that several factors contributed to the evolution of resistance. In particular, continuous planting of maize containing the same Bt trait, coupled with limited adult dispersal, likely favored the evolution of resistance. Additionally, it appears that substantial standing genetic variation for resistance, non-recessive inheritance of resistance, and minimal fitness costs of resistance also contributed to resistance development in the field. 

## 5. Resistance to Bt maize in the Agricultural Landscape 

Initial characterization of Bt resistance focused on fields with high levels of rootworm feeding injury to Cry3 maize (i.e., Cry3Bb1 maize and mCry3A maize) [3,23,38,39,40,41,42,43]. However, this raised the question of how common Cry3 resistance was within the broader agricultural landscape, and how cropping practices might in turn influence patterns of pest abundance and pest injury. 

Landscape-level patterns of Cry3 resistance were examined for western corn rootworm populations in Nebraska by Reinders et al. [5]. This study considered populations at a spatial scale of 2 to 10 Km, and examined two areas of intensive maize production. The authors found significant spatial variation among populations in the level of resistance to Cry3Bb1 maize and mCry3A maize, with a few populations showing no difference in survival from the susceptible controls. This work also looked at the association of various field-history variables with the level of Bt resistance, and found that the use of a Bt trait within a field showed a positive relationship with the level of resistance, which in turn highlights the importance of diversified management in delaying resistance [5]. 

Studies of Cry3 resistance in the agricultural landscape in Iowa include work by St. Clair et al. [6,7]. St. Clair et al. [6] tested for resistance in fields with a history of high levels of feeding injury to Cry3 maize (i.e., past problem fields) and fields that were in close proximity (within < 2.2 km) to past problem fields. This study found that both field types harbored Cry3Bb1-resistant populations of western corn rootworm [6]. Similarly, St. Clair et al. [7] compared counties in Iowa with and without a known history of past Cry3 problem fields. Bioassay data revealed the presence of Cry3Bb1 resistance in both types of counties and found similar levels of resistance. In both studies, there was some variability in the level of resistance, with some populations displaying complete resistance while others had incomplete resistance (i.e., survival or larval development was lower on Cry3Bb1 maize compared to non-Bt maize). These studies suggested widespread resistance to Cry3Bb1 maize in Iowa, although there was variation among populations in the level of resistance. 

In a study examining landscape-level patterns of resistance and the effects of cropping practices on resistance, Shrestha et al. [4] measured Cry3Bb1 resistance in western corn rootworm from fields in Iowa with a variety of management histories, including (1) fields in continuous maize production, (2) rotated fields, (3) past Cry3 problem fields, and 4) current Cry3 problem fields. Data from plant-based and diet-based bioassays illustrated that all field types harbored Cry3Bb1-resistant western corn rootworm [4]. However, larval development on Cry3Bb1 maize was significantly reduced compared to non-Bt maize for rotated fields and past problem fields, but not for continuous maize fields or current problem fields, suggesting that crop rotation may help delay the development of Cry3Bb1 resistance [4]. 

St. Clair and Gassmann [76] analyzed landscape-level patterns of maize cultivation in past problem fields and in areas surrounding past problem fields in Iowa during the timeframe when these fields failures occurred. These patterns were compared with randomly selected agricultural fields in Iowa during the same time period [76]. This study found that, not only were past problem fields characterized by higher levels of continuous maize cultivation compared to randomly selected fields, but also that the local landscape around these past problem fields had more continuous maize than randomly selected points [76]. The local landscapes around past problem fields with elevated percentages of continuous maize cultivation included an area within 3.2 km of past problem fields. Furthermore, available data indicate that 57% of fields in continuous maize production during the timeframe examined contained Cry3Bb1 maize [22]. These studies point to the role of the local landscape in facilitating the evolution of resistance, and suggest that continuous Bt maize cultivation in the broader agricultural landscape contributed to Bt resistance and high levels of feeding injury in past problem fields. 

With the emergence of field-evolved Cry34/35Ab1 resistance, a key question now becomes how widespread Cry34/35Ab1 resistance is within the agricultural landscape [49,50,51]. For Cry3Bb1 resistance, the initial occurrence of Cry3Bb1 resistance in 2009 was followed rapidly by widespread resistance within the agricultural landscape by 2015 [3,4]. As such, characterizing the distribution of Cry34/35Ab1 in the agricultural landscape and taking steps to delay the evolution of Cry34/35Ab1 resistance are of critical importance. 

## 6. Approaches for Improving Resistance Management and for Managing Resistant Populations

The presence of Bt resistant populations within the agricultural landscape raises questions about how best to manage these populations, and how to delay additional cases of Bt resistance. When resistance to Cry3 maize developed, farmers responded by planting maize that contained a pyramid of Cry3 and Cry34/35Ab1 [22]. This approach was effective at mitigating Cry3 resistance, with western corn rootworm population size and root injury scores in these past problem fields returning to levels that were similar to other maize fields in the agricultural landscape [22]. However, resistance to Cry3Bb1 continued to persist in these fields [4]. With the more recent development of resistance to Cry34/35Ab1, there are now western corn rootworm populations that possess resistance to all commercially available Bt traits, and consequently, the challenge of managing western corn rootworm has become more difficult [51]. 

One approach used by farmers to mitigate the effects of Bt resistance has been to combine Bt maize with soil-applied insecticide [6,7,22]. Available data indicate that this approach has short comings both in terms of integrated pest management and insect resistant management. Specifically, if a rootworm population is not resistant to a Bt trait, the reduction in root injury achieved by applying soil insecticide to Bt maize is minimal, and the yield preserved does not appear to justify the cost of the insecticide application [77]. Furthermore, the reduction in survival achieved by adding insecticide does not appear sufficient to provide an effective pyramid with a Bt trait, and therefore, is not expected to delay the evolution of Bt resistance [77,78]. 

Additionally, lessons learned from studies where Cry3Bb1 maize and soil insecticides were combined to manage Cry3-resesitant populations cast light on the general short comings of combining Bt maize and soil insecticide to manage Bt-resistant populations. Shrestha et al. [78] studied Cry3Bb1-resistant populations and tested how the use of Cry3Bb1 maize with soil-applied insecticide affected root injury and survival of western corn rootworm. Applying soil-applied insecticide to Cry3Bb1 maize did not significantly reduce adult emergence compared to the use of Cry3Bb1 maize alone, suggesting that the number of Bt-resistant individuals produced within a field would not be reduced by adding insecticide. Furthermore, the reduction in root injury achieved by combining Cry3Bb1 maize with soil-applied insecticide did not differ from non-Bt maize with insecticide, indicating that farmers did not achieve a benefit in terms of root protection by adding insecticide to Bt maize compared to using non-Bt maize with soil insecticide. These data illustrate that, once a population develops Bt resistance, using soil-applied insecticide with a Bt trait that has been compromised by resistance is not a worthwhile strategy because it will continue to select for resistance, while providing little addition benefit to farmers in terms of preserving yield. 

It is important to note that, when resistance to Cry3Bb1 maize arose, farmers with past problem fields responded by continuing to grow maize, but began using a pyramid of Cry34/35Ab1 with Cry3Bb1 [22]. Because these fields harbored Cry3Bb1-resistant western corn rootworm, the ability of this Bt pyramid to delay resistance was compromised [3,4,23,38,39]. Furthermore, the use of Bt pyramids to manage Cry3-resistant populations likely hastened the evolution of Cry34/35Ab1 resistance [49,51]. The application of a more integrated approach to management of corn rootworm in these fields, including the use of crop rotation and non-Bt maize with soil-applied insecticide, could have helped to delay the development of Cry34/35Ab1 resistance [3,62,79,80]. 

The use of soil-applied insecticide with non-Bt maize reduces selection for Bt resistance, reduces root injury, and permits the survival of corn rootworm to adulthood [77,78,81]. Specifically, the use of non-Bt maize with soil-applied insecticide produces a temporal refuge (i.e., a year in which selection for Bt resistance is absent) thereby enabling the survival of Bt-susceptible individuals in addition to relaxing selection for Bt resistance. In situations where maize is grown for several consecutive years, rotating Bt maize with non-Bt maize that has soil-applied insecticide should both preserve yield and delay the evolution of Bt resistance. This concept is supported by the results of a computer simulation study conducted by Martinez and Caprio [82], which found that use of non-Bt maize with soil-applied insecticide delayed Bt resistance by western corn rootworm. However, it is important to note that the resistance-management benefit of refuges is in delaying resistance [24,28]. Once resistance evolves and is prevalent within a population, refuges of any type (e.g., structured, integrated, naturally occurring or temporal) will be of minimal value in managing resistance. 

Rotating fields out of maize production (i.e., crop rotation) may also aid in reducing Bt resistance and the high levels of feeding injury that can be associated with Bt resistance [4,76,83]. Because maize is the primary larval host for western corn rootworm, crop rotation will eliminate a western corn rootworm population from a maize field, which should increase the ability of farmers to maintain rootworm population below the economic injury level [9]. Work by St. Clair and Gassmann [76] illustrated that high levels of rootworm feeding injury to Bt maize by western corn rootworm, and Bt resistance, were associated with continuous maize cultivation in the local landscape. This study also points to the potential of crop rotation to reduce the occurrence of high levels of feeding injury to Bt maize by Bt-resistant western corn rootworm [76]. Carrière et al. [83] found that increased crop rotation was associated with a reduction in high levels of feeding injury to Cry3Bb1 maize by western corn rootworm. In addition to reducing high levels of feeding injury by Bt-resistant rootworm, Shrestha and Gassmann [4] revealed that crop rotation also can delay the evolution of Bt resistance within a field, an effect that likely arises because of the recolonization of a field by rootworm in neighboring fields. However, the benefit crop rotation in delaying resistance will be contingent on the level of Bt resistance in rootworm populations from neighboring fields, because these populations will serve as a source of recolonizing individuals after a field is rotated back to maize. Work by Reinders et al. [5] highlights the potential for fields to display a high level of Bt resistance following crop rotation if resistance is prevalent in the surrounding landscape. As such, the use of integrated pest management within the broader agricultural landscape is likely to be an important factor influencing the level of Bt resistance in a field following crop rotation. 

The success of future transgenic traits may be affected, in part, by resistance to current Bt traits, particularly in cases where current traits will be pyramided with future traits. The use of RNA interference (RNAi), induced by double-stranded RNA (dsRNA), represents a novel approach for managing rootworm, and DvSnf7 will likely be the first RNAi trait used to manage rootworm [84,85]. However, this RNAi trait will be pyramided with current Bt traits, specifically Cry3Bb1 and Cry34/35Ab1 [86]. Consequently, to the extent that resistance to Cry3Bb1 and Cry34/35Ab1 is present in the landscape, the IRM benefit of pyramiding will be compromised [30]. Widespread resistance to Cry3Bb1 in some regions of the Corn Belt, coupled with emerging resistance to Cry34/35Ab1, may enable the rapid evolution of resistance to RNAi. In a laboratory selection experiment, western corn rootworm was evaluated for resistance to RNAi after seven generations of selection and found to be resistant to multiple insecticidal dsRNA molecules [87]. An alternative approach, where an RNAi trait is coupled with a novel insecticidal protein derived *Pseudomonas chlororaphis*, may provide a more effective IRM approach [88,89]. However, because of the limited dispersal displayed by adult western corn rootworm, diversified management within a field will still be essential to delay resistance to this novel pyramid. Additionally, pyramiding of traits to delay resistance is dependent on the presence of refuges [30]. Because of the limited adult dispersal displayed by adult western corn rootworm, the use of integrated refuges (i.e., blended refuges) with these novel transgenic technologies, should improve mating between resistant individuals and refuge insects, thereby delaying resistance [53,54]. 

Past work has shown that refuges can delay Bt resistance in western corn rootworm [45]. However, widespread field-evolved resistance by this pest also illustrates that refuges alone are not sufficient to delay resistance [4], and fields in continuous maize cultivation with continuous use of the same Bt traits can serve as foci for resistance within the agricultural landscape [3,76]. The use of more integrated pest management, including crop rotation and the use of non-Bt maize with soil-applied insecticide, will be important for delaying additional cases of resistance to current and future transgenic technologies by this serious agricultural pest [90].

## 7. Conclusions

The evolution of resistance to Bt maize by western corn rootworm illustrates the potential for insect pests to develop resistance to Bt crops when a high dose is not present. Key factors that facilitated field-evolved resistance by the western corn rootworm included non-recessive inheritance of resistance and minimal fitness costs of resistance (Table 1 and Table 2). Furthermore, the use of Bt events singly before pyramiding and cross-resistance among Bt traits, likely hastened resistance development [31,32]. The use of novel, pyramided transgenic traits, for which resistance allele frequency is low, should provide a more durable approach for managing this pest [25,30,32].

Another important factor facilitating resistance evolution is the limited dispersal displayed by western corn rootworm adults [52,53]. This factor is intrinsic to the biology of the pest and cannot be manipulated. However, resistance management approaches can be refined to take into account this important aspect of pest biology. Specifically, integrated refuges should be used to increase mating between Bt-selected individuals and those emerging from refuge plants [91]. Additionally, continuous cultivation of Bt maize, coupled with limited adult dispersal, appears to be an important driver of resistance development within the agricultural landscape [3,5,76]. Consequently, more diversified management, including crop rotation and use of non-Bt maize with soil-applied insecticide, should help to delay the development of resistance to current and future transgenic traits for management of western corn rootworm [82,90].

## Figures and Tables

**Table 1:** Studies testing the inheritance of resistance to *Bacillus thuringiensis* (Bt) maize by western corn rootworm.

Type of Resistance ^1^	Strain	Resistant to Toxin ^2^	Type of Assay ^3^	Metric Used	Inheritance of Resistance	Heritability ^4^	Reference
Laboratory Selected	Constant Exposure	CryBb1	Single Plant	Larval Survival	Non-Recessive	0.29	[47]
Laboratory Selected	Constant Exposure	Cry3Bb1	Single Plant	Survival to Adult	Non-Recessive	0.30	[47]
Laboratory Selected	Brookings Moderately Selected	Cry3Bb1	Seedling Mat	Larval Survival	Non-Recessive to Dominant	0.19 to 1.22	[63]
Laboratory Selected	Brookings Moderately Selected	Cry3Bb1	Seedling Mat	Larval Growth	Non-Recessive	0.51	[63]
Laboratory Selected	mCry3A selected	mCry3A	Single Plant	Larval Survival	Non-Recessive	0.66	[58]
Laboratory Selected	mCry3A selected	mCry3A	Single Plant	Survival to Adult	Dominant	1.03	[58]
Laboratory Selected	eCry3.1Ab selected	eCry3.1Ab	Seedling Mat	Larval Survival	Dominant	0.94 to 1.38	[64]
Field Evolved	Hopkinton	Cry3Bb1	Seedling Mat	Survival to Adult	Non-Recessive	0.37	[65]
Field Evolved	Cresco	Cry3Bb1	Seedling Mat	Survival to Adult	Recessive	0.27	[65]
Field Evolved	Elma	Cry3Bb1	Seedling Mat	Survival to Adult	Non-Recessive	0.14 to 0.29	[66]
Field Evolved	Monona	Cry3Bb1	Seedling Mat	Survival to Adult	Non-Recessive	0.45	[66]
Field Evolved	Monona	Cry3Bb1	Single Plant	Larval Survival	Non-Recessive	0.73	[66]
Field Evolved	Monona	Cry3Bb1	Diet Based	Larval Survival	Non-Recessive	------ ^5^	[66]
Field Evolved	Central Iowa	Cry3Bb1	Single Plant	Larval Survival	Non-Recessive	0.23	[67]
Field Evolved	Eastern Iowa	Cry3Bb1	Single Plant	Larval Survival	Non-Recessive	0.50	[67]
Field Evolved	Northern Iowa	Cry3Bb1	Single Plant	Larval Survival	Non-Recessive	0.54	[67]
Field Evolved	Western Iowa	Cry3Bb1	Single Plant	Larval Survival	Recessive	0.08	[67]

^1^ Describes whether a strain was generated by selecting a susceptible stain on Bt maize in the laboratory (Laboratory Selected) or by collecting Bt-resistant insects from the field (Field Evolved). ^2^ Type of Bt maize on which a rootworm strain was selected and to which it was resistant. ^3^ Describes the bioassay approach that was used to measure resistance. Details are provided within the references, but in general, these approaches involved measure survival on single plants in containers (single-plant assay), on a mat of maize roots generated by germinating a small number of maize seeds in a container (seedling-mat assay) or in an assay where Bt toxin was placed on top of an artificial diet (diet-based assay). ^4^ Heritability is a metric that describes the extent to which heterozygous individuals resemble the parental strains (i.e., Bt resistant and Bt susceptible) for survival on Bt maize. Specifics on each calculation are given within individual references, but in general, a score of 0 indicates equal survival on Bt maize between the parental Bt-susceptible strain and heterozygotes, 1 indicates equal survival on Bt maize between the parental Bt-resistant strain and heterozygotes, and 0.5 indicates that survival of the heterozygotes on Bt maize is halfway between that observed for Bt-resistant and Bt-susceptible parental strains. Scores greater than 1 occur when the heterozygotes have higher survival on Bt maize than their parental Bt-resistant strain. ^5^ Heritability was not calculated because an LC_50_ for the resistant strain could not be determined.

**Table 2:** Studies testing for fitness costs of resistance to Bt maize by western corn rootworm.

Type of Resistance ^1^	Strain	Resistant to Toxin ^2^	Cost Present? ^3^	Traits Affected ^4^	Reference
Laboratory Selected	Brookings Moderately Selected	Cry3Bb1	No	-----	[63]
Laboratory Selected	Brookings Moderately Selected (Strain 1)	Cry3Bb1	No	-----	[71]
Laboratory Selected	Brookings Moderately Selected (Strain 2)	Cry3Bb1	No	-----	[71]
Laboratory Selected	Brookings Moderately Selected (Strain 3)	Cry3Bb1	No	-----	[71]
Laboratory Selected	Brookings Intensely Selected (Strain 1)	Cry3Bb1	No	-----	[71]
Laboratory Selected	Brookings Intensely Selected (Strain 2)	Cry3Bb1	No	-----	[71]
Laboratory Selected	Data Presented as Composite of Three Resistant Strains	CryBb1	Yes	Fecundity; Adult (male) Longevity	[68]
Laboratory Selected	Brookings Moderately Selected	CryBb1	No	-----	[70]
Laboratory Selected	Brookings Moderately Selected	CryBb1	Yes	Larval Development; Egg Viability	[69]
Laboratory Selected	mCry3A Selected	mCry3A	No	-----	[58]
Laboratory Selected	eCry3.1Ab Selected	eCry3.1Ab	No	-----	[72]
Field Evolved	Hopkinton	Cry3Bb1	No	-----	[65]
Field Evolved	Cresco	Cry3Bb1	Yes	Larval Development; Survival to Adulthood; Fecundity	[65]
Field Evolved	Elma	Cry3Bb1	Yes	Larval Development	[66]
Field Evolved	Monona	Cry3Bb1	No	----	[66]
Field Evolved	Cresco	Cry3Bb1	Yes	Decline in Resistance over Time	[73]
Field Evolved	Hopkinton	Cry3Bb1	Yes	Decline in Resistance over Time	[73]
Field Evolved	Data Presented as Composite of Eight Resistant Strains	Cry3B1	Yes	Adult Size	[67]

^1^ Describes whether a strain was generated by selecting a susceptible stain on Bt maize in the laboratory (Laboratory Selected) or was generated from Bt-resistant insects collected from the field (Field Evolved). ^2^ Type of Bt maize on which the rootworm strain was selected and to which it was resistant. ^3^ States whether fitness costs of Bt resistant were detected for a specific strain in a study. ^4^ Life-history traits for which a fitness cost was detected or cases where resistance declined over time when a stain was not exposed to Bt maize.
